# Vaccine hesitancy: Report of a student study group

**DOI:** 10.1007/s00508-020-01655-4

**Published:** 2020-04-22

**Authors:** Lisa Weitz, Luise Bellach, Alicia Faltum, Angelika Berger, Wolfgang Maurer

**Affiliations:** 1 Medical University of Vienna, Vienna, Austria; 2 Division of Neonatology, Pediatric Intensive Care, and Neuropediatrics, Comprehensive Center for Pediatrics, Medical University of Vienna, Waehringer Guertel 18-20, Vienna, 1090 Austria

**Keywords:** Vaccine hesitancy, Vaccine sceptics, Anti-vaccine groups

## Abstract

In summer 2019 an extracurricular activity was started at the Medical University of Vienna (MUW) with the title: “Esoterism in Medicine”, where different chapters were evaluated by students. Here we present the subheading “Vaccine Hesitancy”. Three students formulated arguments from sceptic, hesitant or anti-vaccine groups and discussed the scientific literature to rebut it. Frequent objections were partly taken from the homepage of the German Robert-Koch-Institute, the home of the “Ständige Impfkommission”. Other objections were taken from blogs and social media. The students’ rebuttal was based on current scientific literature (preferentially pubmed), but also from other scientific sources like authorities.

## Introduction

The European Pharmacopoiea defines vaccines for human use as follows: “Vaccines for human use are preparations containing antigens capable of inducing a specific and active immunity in man, against an infective agent or the toxin or antigen elaborated by it” [1]. Medicinal products sometimes named as therapeutic vaccines do not comply with this legal definition.

Medicinal products can be divided into pharmaceuticals and biologicals, such as stable blood products, monoclonal antibodies, interleukins and vaccines. Vaccines are the most complex medicinal products and cannot be characterized by normal analytical tests. A consistent manufacturing process is important for their characterization. The European Medicines Agency (EMA) states that “vaccines are complex biological medicinal products. Currently, it seems unlikely that these products may be thoroughly characterized on a molecular level” [2]. Vaccines are most important for public health but have high complexity as products and also are highly regulated by authorities in the production process [3]. New vaccines in the EU have to be licensed by the EMA, the procedure includes inspections (also unannounced) of the manufacturing plant, quality systems, quality and origin of raw materials, production plant. The application for marketing authorization includes all preclinical and clinical data. If a vaccine is licensed, each batch (of vaccine produced in one production run) in addition to the manufacturing batch release procedure is again tested for conformance by an independent state Official Medicines Control Laboratory (OMCL).

Each vaccination reduces the probability of a specific infection depending on its efficacy. Adverse events can follow. These can be causal, such as pain, swelling or redness. For other adverse events following immunization (AEFI) causality is often not clear. Most other AEFIs are only coincidental after immunization and are acausal; however, because of the temporal association, the general public may believe that a certain event is causally related. In clinical trials before licensing, every event post-vaccination is recorded (including traffic accidents), and an investigation is done if an event is considered to be causal. Such a procedure is not possible in normal life but in the population risk assessment underestimates the benefit of vaccination and overestimates the risk of immunization.

Vaccine hesitancy is a global threat against global health; according to WHO it refers to “delay in acceptance or refusal of vaccines despite availability of vaccine services” [4]. The range of vaccine hesitancy is broad including hardcore anti-vaccine groups. Anti-vaccinationists discuss the right time for a vaccination, they have questions about vaccine safety or about the strategy of immunization. Some of them do not approve of certain vaccines, such as those against multiple diseases in one syringe but some can sometimes be convinced to accept immunizations, while some prefer individualized approaches.

Few people in vaccine hesitancy groups (“anti-vaxxers”) are generally completely against immunization. Frequently, they use irrational reasons: “a so-called measles virus does not exist” [5], or a physician (!) claiming that “nobody has seen this hypothetical (rabies) virus” [6]. Within this movement are people who are against vaccination for religious reasons, such as those living in the Dutch Bible Belt. There was a rubella outbreak there among pregnant women, resulting in two fetal fatalities and 14 infants with congenital rubella with multiple symptoms, including deafness [7].

An anti-vaccine movement has existed since smallpox vaccination started. In the 1880s a journal was founded entitled *Against Compulsory Vaccination. Monthly Journal for Public Health and Heresy of Physicians* [8]. Its claims were refuted in other journals [9]. The anti-vaccine movement has been marked by scientific hostility and irrationality since the nineteenth century. *Belief in pathogenic germs and the obsessive belief in witches are products of the same mind-set* [10]. Although such mind-sets were not very widespread in the pre-internet era, nowadays irrational belief of the anti-vaccine movement can spread very quickly on social media. On a German website *impfkritik.de*, the human immunodeficiency (HIV) virus is rejected as the initial cause of AIDS, the existence of a measles virus is denied; it also states that the former use of the insecticide DDT is the true cause of poliomyelitis.

Frequent assertions were partly taken from the homepage of the German Robert Koch Institute, Standing Committee on Vaccination [11]. Other assertions were taken from blogs and social media. The students’ rebuttal was based on current scientific literature (preferentially the NIH-based public scientific database PubMed), but also from other scientific sources such as the Paul Ehrlich Institute. The results of the students’ work are presented.

## Prognosis, mortality and morbidity of two vaccine preventable diseases

### Influenza vaccination

Due to the mutation of circulating wild-type influenza virus it is necessary to propose influenza vaccine sequences for the next influenza season. This recommendation takes place in February or March by the WHO and the EMA, based on the results of sequencing wild-type isolates worldwide; however it is not possible to predict which of four influenza strains will dominate within a season.

#### Assertion

Vaccine hesitants argue that influenza vaccination is of marginal efficacy and harmful due to the ovalbumin content.

#### Rebuttal

Although different vaccines against influenza infections are available, it is true that the efficacy of influenza vaccines is limited. In addition, vaccine efficacy can vary during the influenza season, when circulating strains mutate [12]. Vaccine coverage in Austria is dramatically low—an estimate is 6% of the general population [13]. Austria has a population of about 8.9 million. Influenza-associated fatalities peaked during some years between 2001–2009, with 1060 fatalities in 2002/2003, 1102 in 2004/2005 and 1102 in 2008/2009. This high rate is coincident with the increasing age of the population but may also be due to the low vaccine uptake [14]. These influenza-associated cases are twice as high as the deaths due to road traffic accidents (2016: 432 and 2017: 414 cases).

During the swine flu pandemic in 2009 in the USA—the H1N1 influenza pandemic—the CDC (The American Center for Disease Control and Prevention) received 788 reports of pregnant women affected by the virus—30 of them died. Among 509 hospitalized women, 115 (22.6%) were admitted to an intensive care unit (ICU). In the USA the pandemic occurred outside the normal influenza season, and it can be assumed that all pregnant women were immunologically naive to the new H1N1 [15]. In Austria, influenza vaccination is recommended for everyone from the age of 7 months by the Ministry of Health. In addition, the flu vaccine is strongly recommended for pregnant women and those wanting to get pregnant in the next influenza season.

The ovalbumin content of influenza vaccines is a residue of production when fertilized eggs are used, and is no longer considered a contraindication, with the exception of children with a severe egg allergy including anaphylaxis and ICU treatment [16]. Influenza viruses grown on other cells is also available.

### Measles vaccination

According to the WHO, the European Union (EU)/EEA (European Economic Area) member states pledged to eliminate measles from the European region by 2000. This plan was not successful and was initially extended to 2010 and then to 2015 [17]. The WHO objectives for elimination have still not been met in 2019 in the EU. In Austria, 77 cases were reported in 2018, but even more in 2019 and by October 16 there were 146 reported cases. In 2018 more than 80,000 cases were reported in the WHO European region, including 72 fatalities [18]. That elimination of measles is possible was shown in the WHO region of The Americas, which was declared free of measles in September 2016 [19], but a new outbreak started in August 2017 in Venezuela, in the meantime covering the whole WHO region with 6573 confirmed or suspected cases by October 2019 [20]. While measles is not eradicated globally, the only possibility of reaching herd immunity to stop the circulation of measles virus is two measles immunizations with ≥95% vaccine coverage.

#### Assertion

The MMR vaccine causes autism [21].

#### Rebuttal

This anti-vaccine assertion is attributed to a study by Wakefield et al. in 1998 [22]. The study suggested that children who had the MMR vaccination were more likely to develop behavioral disorders including autism. The trial included 12 children (11 boys, 1 girl), in the age range 3–10 years. All children underwent a series of medical assessments. From a scientific point of view the study was not of high quality. There was no control group and the cohort was too small to show a significant outcome. Despite that, the paper was published in The Lancet. In 2004 The Sunday Times revealed evidence that a solicitor sponsored Wakefield to find a link between vaccines and autism [23]. This caused the retraction of the publication in 2010 and all co-authors except Wakefield resigned their support from the trial’s interpretation [24]. Wakefield’s medical license was removed [25]. He is currently living in the USA and still canvassing his anti-vaccine beliefs. One of those arguing that vaccination causes autism was President Trump (USA), who tweeted on 28 March 2014: *healthy young child goes to doctor, gets pumped with massive shot of many vaccines, doesn’t feel good and changes—AUTISM. Many such cases!* The American Academy of Pediatrics responded to this in a letter signed by 350 scientific organizations. In an appendix, they added 40 references that neither the MMR vaccine nor thiomersal are causally related to autism [26].

In the following years many systematic reviews were done, which proved that there is no causality between the MMR vaccine and autism [27]. The systematic review by Demicheli et al., which included 14,700,000 children, found a significantly higher risk of febrile seizure after MMR vaccine exposure, but could not find any association between the vaccine and autism [28]. But there is still a lot of misinformation, especially through social media, which leads to vaccine hesitancy [29].

#### Assertion

Maternal passive immunity from mothers after natural infection with measles lasts for 10 months, vaccine-induced immunity only for 3–6 months. Therefore, there is a higher risk of infants <10 months getting measles [30], which is most severe in the first year of life.

#### Rebuttal

Newborns born at term lose 50% of measles antibodies every 21–28 days. These antibodies protect from measles disease, but not from measles infection. These infants have no measles symptoms (asymptomatic measles or no typical symptoms) but they can be infected [31]. In healthy children, all exposed to wild-type measles, the virus was found in lymphocytes of RT-PCR positive infants. No virus was found in palatine tonsils, suggesting that these asymptomatic children were infected but not infectious [32]. Measles in the first year of life has the highest mortality and the highest risk (1:600 infected) of developing deadly subacute sclerosing panencephalitis (SSPE). Before 2002 it was found that 50% of SSPE cases were diagnosed in children <2 years old [27].

To avoid wild-type measles infections in the first year of life, herd immunity is necessary, which means interruption of measles virus circulation, achieved by immunization of >95% of all non-immune persons.

In the past, without availability of antibiotics (against bacterial superinfections) and pediatric intensive care units, measles infections were much more severe. Although robust statistical data are missing, measles mortality was extreme in the first year of life (about 25%) and >95% of measles deaths occurred below the age of 15 years. In total it was estimated that 3% of a birth cohort died of measles in Europe 100–120 years ago [33].

### Vaccinations are the most important preventive measure against infectious diseases

#### Assertion

The decline of infectious diseases was due to better hygiene and nutrition.

#### Rebuttal

Another assertion of vaccine opponents is that the reason for the decreasing number of infections was not caused by introducing new vaccines, instead it was because of better hygiene and nutrition [34, 35]. There was a great improvement in quality of hygiene and nutrition in the last century. To understand the correlation between these two parameters and their impact on human lives it is best to look at life expectancy. Life expectancy shows a steady, linear gain. In the year 1880 it was below 40 years and in 2015 it was twice as high [36]. The numbers of reported measles cases however decreased exponentially after the nationwide introduction of the measles vaccine in 1963 [37–39]. In the 1920s, approximately 3% of a birth cohort died of measles in early life [33]. The same decrease happened with poliomyelitis. In the 1950s, two polio vaccines were developed and came on the market. Jonas Salk developed a vaccine that protected the central nervous system and a few years later Albert Sabin introduced an oral live vaccine which also prevented transmission and infection through the digestive system. Over 350,000 paralytic poliomyelitis cases were estimated globally in 1980. In 1988, the Global Polio Eradication Initiative was founded, resulting in a further decrease of paralytic polio, with 42 cases world-wide in 2016 [40]. In 2019 only two countries reported polio cases (Pakistan, Afghanistan). The switch from endemic early asymptomatic polio infection to epidemic outbreaks of polio can also be explained by better hygiene. In the nineteenth century hygiene standards were far lower and children were exposed to the polio virus very early in life while still having a passive immunization through their mother. Later, in the twentieth century, the infection developed at a later point in life without protection of passive antibodies, causing epidemic outbreaks. After establishing a nationwide vaccination program in the USA, the numbers of poliomyelitis cases dropped from 57,879 in 1952 to 910 in 1962. Herd immunity was reached due to high coverage and The Americas were declared free of polio in 1994 [41].

#### Assertion

Infections are not so bad, they can be treated. We, the parents, had overcome them without vaccination.

#### Rebuttal

This argument has a severe bias, because those who died of infections early in life are not able to become parents.

Not only do infections like measles and human papilloma virus (HPV) have a poor prognosis, they can also cause other diseases as a complication. The background of this argument is on the one hand the trust of parents in state of the art medicine and on the other hand less public awareness of complications of the infection. Measles has the following complications: otitis media, pneumonia, postinfectious encephalomyelitis, SSPE and sustainable immunosuppression [42, 43]. The mortality of measles nowadays is 1 in 1000 cases in industrialized countries. The two most severe complications are SSPE and sustainable immunosuppression. SSPE has an incidence of 1 in 5000–10,000 but is even more common when infection occurs in the first year of life (1:600). The reason why SSPE is a dangerous complication is because there is no cure and it will inevitably lead to death. Herd immunity is necessary to protect from SSPE. When measles was eliminated from the USA, SSPE decreased to zero for children born in the USA.

Sustainable immunosuppression is caused by the continuous destruction of memory cells. It increases the risk of being infected and dying from other non-targeted diseases [44].

The most common sexually transmitted disease is HPV. There are low-risk and high-risk virus types. The low-risk types are not carcinogenic but can lead to genital warts. The high-risk types are carcinogenic [45–47]. A persistent infection can lead in 10% of cases to cancer of the cervix, vulva, anus, penis and oropharynx. Cervical cancer in particular has a high incidence and >95% are caused by HPV. A meta-analysis has shown that in high income countries 5–8 years after HPV vaccination the prevalence of HPV 16 and 18 decreased by 83% in girls aged 13–19 years [48]. HPV vaccines are highly effective and prevention is the better choice over treatment.

#### Assertion

The pharmaceutical industry will only make huge profits with vaccines.

#### Rebutta

In Austria vaccine costs cannot easily be calculated. Many of the generally recommended vaccines of the national vaccination plan (*Impfplan*) have to be paid for privately (varicella, mengicoccal B, influenza, TBE vaccines). Some pediatric vaccines are free of charge, such as hexavalent, pneumococcal, HPV, MMR vaccines. They are bought on tender from the Ministry of Health, however the vaccine prices in this tender are not published in Austria. In addition, some federal states give additional allowances to certain vaccines.

In general, beyond the age of compulsory school attendance, all vaccines have to be paid for privately at pharmacy prices.

In Germany statutory health insurance covers 90% of insured persons. The Robert Koch Institute (RKI) states about vaccine costs: of nearly 194 billion € spent by insurance companies in 2014, 33 billion (17%) were spent on medicinal products and only a little over 1 billion € (0.65%) was spent on vaccines; however, normal medicinal products have to be given frequently (e.g. insulin) whereas vaccines are needed infrequently (such as MMR twice over a person’s life). Since production of vaccines is costly and complex [49], there are only a handful of vaccine producers worldwide.

With few exceptions, most vaccines are cost-saving, particularly when also considering the additional indirect protection of unvaccinated people due to herd immunity. For economic assessment the term number needed to vaccinate to prevent one case/one hospitalization/one death (NNV) is used. In this connection only the vaccinated group is calculated, but not other age groups who are indirectly protected due to reduced frequency of infection or herd immunity.

The driving force behind infection through influenza is children <6 years. They can infect others (grandparents!), therefore by vaccinating children, older age groups have a reduced risk of infection [50].

Although in oncology physicians cannot be responsible for prices of anti-cancer drugs, the benefit of new anti-cancer drugs may only last some months, but for a high price. As an example, the treatment of late-stage colorectal cancer with bevacizumab has a price tag of 50,000 $ per treatment episode, but only a benefit of incremental increase of life expectancy of 5 months. On average 9 treatment episodes are necessary [51].

Prevention programs may provide a cost saving. But what is the price? In road safety in the EU a fatality avoided by introducing safety measures was worth 1 million € (the one million € rule introduced by the European Commission in 1997 based on calculations of 1995). Two improvements were made, resulting in acceptable costs per fatality avoided of 4.05 million €—this results in acceptable costs of about 5 million € nowadays [52]. In contrast, to avoid one fatality from cervical cancer, only 101 girls (aged 9–14 years) need to be immunized with HPV vaccines. Assuming tender prices of 55 €/dose and a two-dose regimen, costs are less than 11,100 € to avoid one female death caused by HPV [53]; however, in Austria the recommendation is to immunize boys and girls: a whole birth cohort (*n* ~ 80,000) has therefore to be vaccinated with costs of about 8,800,000 € to reach the prevention optimum, including genital warts and male cancer. About 300 women die each year from cervical cancer [54], so one can make a rough estimate of vaccine costs of 30,000 € to prevent one woman’s death from oncogenic HPV. Hence this is significantly cheaper than costs of death prevention in road traffic accidents.

A gender aspect can be also discussed since about 74% of deaths in road traffic are men but 100% of cervical carcinoma occur in women.

#### Assertion

Vaccination is an individual decision

#### Rebuttal

The principle of so-called herd immunity or population immunity is the evidence-based rebuttal of this argument. Herd immunity is an indirect effect of vaccination and is defined as the indirect protection of unvaccinated people. An increasing number of vaccinated individuals in a population leads to reduced pathogen spreading and therefore to an interruption of the natural chain of infection. Herd immunity and its importance was first noticed with smallpox. The goal was to immunize 80% of the population in order to achieve herd immunity. The eradication of smallpox in 1977 and the declaration by the WHO in May 1980 that “the world and all its peoples have won freedom from smallpox”, was the result of even higher vaccine uptake rates and led to the reduction of smallpox by a mass vaccination program in endemic countries [55]. In a population, immunity can be achieved in two different ways: vaccination or infection. Considering two different subpopulations, the immunized population has to be larger than the one with susceptible individuals. The number of susceptible hosts has to be reduced to a level that interrupts transmission of the pathogen. The necessary vaccine coverage to achieve herd immunity is pathogen-dependent [56]. This is particularly important for individuals who cannot receive any vaccination, such as newborns, immunosuppressed children, or immunologically naïve persons, who all profit from the indirect protection of herd immunity [57]. For example, an unvaccinated woman can profit from the HPV vaccination of her male partners since the high-risk HPV strains 16 and 18 are responsible for 70–80% of cervical cancers.

#### Assertion

Vaccines are dangerous and adverse events cannot be calculated

#### Rebuttal

Like any other medicinal product administered to humans, vaccines have to withstand multiple stages of thorough testing (preclinical and clinical) before they are approved to be used in the general population [58]. International standards for vaccine safety assessment and presentation of the subsequent test results are set by the WHO [59]. During development of a new vaccine, preclinical tests have to be carried out. The preclinical phase consists of testing, determining and specifying the antigen as well as selecting the adjuvants and performing in vitro [60, 61], in silico [62] and in vivo [59] experiments. The preclinical phase, which is subjected to constant ameliorations and updates [63], demands years of research [62]. If this phase is successful, clinical studies must be performed (phases I–III) including up to 60,000 volunteers. If successful, the manufacturer can apply for marketing authorization at the EMA. If the vaccine is successfully approved, a phase IV clinical study follows to detect possible rare AEFI [64, 65].

Each licensed vaccine is required to have a positive benefit-risk ratio. As an example, the risk of death from measles in industrialized countries with intensive care units is 1:1000 measles cases. The incidence of febrile seizures can be as high as 8% and can develop into encephalitis (1–2 per 1000 measles cases); however, measles vaccination cannot cause death if contraindications are considered. Febrile seizures happen in about 1 in 3000 vaccinees, but do not have consequences. It could not be shown that a licensed measles vaccine can cause encephalitis. Adverse events caused by immunization are known as well as complications of the disease averted by immunization.

Background morbidity like headaches, fever or rare cases of invagination [63, 64] will happen in a certain percentage of the population regardless of whether a medicinal product is administered. It was found that during 1 year, one fifth of the population (20.1%) experiences an episode of headache [63].

In order to correctly distinguish between a causal adverse event of immunization or a coincidental acausal event, it is necessary to know the background morbidity of the population [65].

#### Assertion

Vaccines contain poisonous ingredients

#### Rebuttal

Although vaccines have contained adjuvants for decades, anti-vaxers describe aluminium and mercury as poisonous ingredients in vaccines, claiming that aluminium supposedly leads to severe neurological disease. Adjuvants are agents used to enhance the immune response in inactivated vaccines to achieve protection against the pathogen. Raised immunogenicity can be considered a result of four different mechanisms:

1. Depot effect, which develops at the injection site and leads to slow release of antigen.
2. Stabilization of the epitope conformation.
3. Stimulation of macrophages and consequently, activation of lymphocytes.
4. Activation of the complement system.

By adding adjuvants such as aluminium salts, the antigen concentration in vaccines can be reduced as well as the number of required doses in the vaccination schedule [66]. This may be important in increasing the supply of vaccines in new global influenza pandemics.

Aluminium salts are added to vaccines in different formulations such as Al hydroxide, Al hydroxyphosphate, Al phosphate, or Al potassium sulfate. The Al adjuvants are nearly insoluble, and parts may remain as depot in the muscle. Adjuvants are excreted via the kidneys. As it is considered to be effective and safe, vaccines against tetanus, hepatitis A, hepatitis B, human papillomavirus and *Haemophilus influenzae* b include aluminium salts [67]. In Europe, the maximum aluminium amount (calculated as Al^3+^) in one vaccine dose is 1.25 mg, which is significantly less than our aluminium intake through diet or water intake, even if less than 1% of the oral burden is absorbed by the body. In reality the possible maximum aluminium concentration in vaccines is not achieved. Although high aluminium doses can be toxic (concentration maximum in food 2 mg/kg BW per day), there is no evidence supporting teratogenicity or carcinogenicity [66]. There is no difference when comparing aluminium levels of vaccinated subjects with unvaccinated individuals owing to the small quantity of aluminium contained in vaccines [67].

It is important to keep in mind that aluminium salts are frost-sensitive, meaning that at freezing temperatures, aluminium salts can lead to diminished immunogenicity and also to an increase in adverse local reactions. Frozen adjuvanted vaccines must be discarded.

Ethylmercury, also referred to as thiomersal, was added to vaccines as a preservative in multidose containers. [68]. But with the availability of single-dose containers or ready to use syringes, the addition of a preservative was no longer necessary. The fact is that all vaccines for children have been free of thiomersal as preservative since 2000, with the exception of some brands of H1N1 pandemic influenza vaccine in 2009/2010. In Austria, only a preservative-free H1N1 vaccine was used in a 10-dose container during the pandemic. Since live vaccines must not contain a preservative, but are widely used in 10-dose containers, it is questionable whether preservatives are in general necessary in vaccines. In some vaccines thiomersal was also used for inactivation of the antigen. This residue of production remained in some vaccines at less than 1% of the concentration of thiomersal used as a preservative until 2008; however, some anti-vaxers still believe in its toxicity in vaccines, although this topic has been rebutted repeatedly.

Since 1999, the use of thiomersal-containing vaccines has been decreasing worldwide, while the prevalence of autism spectrum disorders is rising. There is no contraindication for the use of thiomersal-containing vaccines in infants, children and non-pregnant women [69]; however, thiomersal is not necessary in single-dose containers (ready to use syringes), when GMP are established.

#### Assertion

Why should I expose my child to a vaccine risk, when the infection rate is practically zero in the EU?

#### Rebuttal

Another common argument used in the community of vaccine hesitancy is that since the initial risk of catching the disease is low, vaccinations against this infectious agent are unnecessary; however, this argument has only limited applicability. While there has been one case in which an existing vaccination program was abandoned because of successful eradication of the causative organism, multiple generations had to be vaccinated in order to make this goal achievable.

Historically, the extensive and consistent vaccination campaigns against smallpox led to complete eradication of the virus in 1980—since then smallpox is dead. [70, 71]. This was possible since the smallpox virus has only one reservoir—humans [72]. Therefore, eradicating pathogens which have more than one host, for example the influenza virus [73], is not possible.

The next disease to have pre-eradication status is polio. Too low vaccination rates in Pakistan and Afghanistan are still enabling the polio virus to spread and caused 20 symptomatic cases of polio in 2016 [74], which increased to 69 reported cases as of August 2019 [75].

It was shown in Israel in 2013 that in sewage 136 samples were positive for poliomyelitis virus, but no infections were found in the population due to high vaccine coverage [76].

Despite the apparent difficulty of poliomyelitis eradication, it has already been proven that via resolution and extensive international collaboration this task is not a utopic experiment but rather a highly challenging yet achievable goal. The proof of principle that poliomyelitis can be eradicated is documented by the disappearance of poliomyelitis type 2 globally, with the last wild-type isolated in 1999.

Global eradication is technically possible for any pathogen that is restricted to replicate or reproduce only in humans; however, this theory has one serious constraint in its feasibility. In order to effectively eliminate any pathogen restricted to a human reservoir, it is crucial to get vaccination coverage as high as possible [77]—the fewer unvaccinated people in a community, the less chance for the pathogen to spread from person to person.

There are always individuals who are too young to be vaccinated or cannot be vaccinated due to clinical reasons, such as underlying medical conditions like HIV infection, leukemia, ongoing chemotherapy, congenital disorders of the immune system or a history of severe reactions against the vaccine in question [78].

Since these individuals cannot get vaccinated and, in many cases, already have to cope with an impaired immune system, they strongly rely on the immune community around them. This concept can be subsumed under the term herd immunity [79, 80].

Therefore, non-medically exempt people refusing to vaccinate can benefit from reduced infectious pressure or herd immunity [81] while at the same time weaken the overall security for those who need to be protected due to the resultant gaps in immunization barriers [82]. Besides the protection of the defenseless non-vaccinable minority, the second pivotal aspect of achieving a high vaccination rate is the possibility of eradicating the pathogen in question, a subject that has already been addressed.

Like the smallpox vaccine regimen, vaccination programs against other organisms replicating solely in humans can be abandoned once this goal is achieved. A prominent example of a human-specific pathogen is the measles virus. Since the measles virus exhibits a high rate of replication [77] and is highly contagious [79, 82], an especially high vaccination coverage is required to regionally eliminate and later eradicate the disease [83]. According to the WHO, 95% of the non-immune population needs to be vaccinated twice against measles to completely stop it from spreading [78]; however, eliminating the virus in just one country and subsequently pausing the vaccination program will not suffice since it would just need one contagious person visiting from a country where the virus has not been eliminated to set off the next measles outbreak if the person was in contact with an unvaccinated individual. To effectively eradicate a human-specific pathogen like the measles virus, worldwide cooperation is needed. Lastly, another obvious yet decisive aspect of vaccinating against diseases like the measles virus is reducing the chance of contracting the disease and any of its entailing complications and keeping secondary risks to a minimum [77]. Thus, vaccinating as many individuals as possible, given that there are no contraindications, maximizes the chances of pathogen eradication and, subsequently nullifies the need for vaccinating against the pathogen in question, as well as minimizing the risks of catching serious vaccine-preventable diseases.

#### Assertion

So many vaccines overwhelm the immune system of my small baby; a hexavalent vaccine is too much for my baby.

#### Rebuttal

Whole cell pertussis (whooping cough) and whole smallpox virus vaccines, albeit characterized by a positive life-saving to risk ratio, have in the meantime become obsolete.

The old whole cell pertussis vaccine contained all carbohydrates, DNA, RNA of a pertussis bacteria, and about 3000 proteins. The smallpox vaccine was a whole virus vaccine; smallpox virus is a huge virus with 200 proteins. A hexavalent vaccine against diphtheria, tetanus, pertussis, poliomyelitis, *Haemophilus influenza* b and hepatitis B infections contains about 23 proteins as antigens. Various brands are licensed. See Table 1.

**Table 1:** Nr of Immunogenic Proteins and Polysaccharides Contained in Vaccines (adapted according to Offit [84])

1900		1960		1980		2015	
Vaccine	Protein	Vaccine	Protein	Vaccine	Protein	Vaccine	Protein/polysaccharides
Smallpox	≈200	Smallpox	≈200	Diphtheria	1	DTaP	5
*Total*	*≈200*	Diphtheria	1	Tetanus	1	Human Papilloma Virus	4–9
–		Tetanus	1	Whole cell Pertussis	≈3000	Rotavirus	65
–		Whole cell Pertussis	≈3000	Polio	15	Polio	15
–		Polio	15	Measles	10	MMR	24
–		Tuberculosis	4000	Mumps	9	Men C	2
–		*Total*	*≈7217*	Rubella	5	HIB	2
**–**				*Total*	*≈3041*	VZV	69
**–**						Pneumococcus	10–23
**–**						Hepatitis B virus	1
**–**						Influenza	6–33
**–**						*Total*	*203–248*

*DTaP* diphtheria, tetanus, and acellular pertussis, *MMR* measles, mumps, rubella, *MenC* meningococcal C, *HIB* Hämophilus influencae type B, *VZV* varicella zoster virus

If one looks at the biological diversity on a childrens playground, 1g of earth contains between 6400 and 38,000 bacterial species (excluding the number of viruses). Since one bacterial species has about 3000 proteins potentially acting as antigens, this amounts to a total of 19.2–114 million antigens [83]. This huge number does not overwhelm our immune system if earth is swallowed or contaminates a small wound. In the 1960s recommended vaccines had a burden of over 7000 antigens, with the BCG vaccine containing about 4000 antigens, administered in the first week of life. Today all generally recommended vaccines have approximately the same number of antigens as were contained in the smallpox vaccine (200 antigens), but we can protect our children from many more infectious diseases.

## Conclusion

The outcome is that students not experienced in vaccinology can clearly rebut assertions of all versions of vaccine hesitancy from sceptical private experience to unfounded opinions circulating in the internet.

## Abbreviations

AEFI: Adverse event following immunization
BCG: Bacillus Calmette-Guérin vaccine against tuberculosis
BSE: Bovine spongiform encephalopathy
DDT: Dichlorodiphenyltrichlorethane, insecticide
DTaP: Diphtheria-tetanus-acellular pertussis vaccine
EMA: European Medicines Agency https://www.ema.europa.eu/en
GMP: Good manufacturing practice
HIB: Haemophilus influenza B vaccine
ICU: Intensive care unit
Men C: Meningococcal C vaccine conjugated
MMR: Measles-mumps-rubella vaccine
MUW: Medical University of Vienna Austria
OMCL: Official Medicines Control Laboratory
RT-PCR: Reverse transcription polymerase chain reaction
SSPE: Subacute sclerosing panencephalitis
TBE: Tick-borne encephalitis
VZV: Varicella zoster vaccine
